# Distinguishing Low-Risk Luminal A Breast Cancer Subtypes with Ki-67 and p53 Is More Predictive of Long-Term Survival

**DOI:** 10.1371/journal.pone.0124658

**Published:** 2015-08-04

**Authors:** Se Kyung Lee, Soo Youn Bae, Jun Ho Lee, Hyun-Chul Lee, Hawoo Yi, Won Ho Kil, Jeong Eon Lee, Seok Won Kim, Seok Jin Nam

**Affiliations:** Division of Breast and Endocrine Surgery, Department of Surgery, Samsung Medical Center, Sungkyunkwan University School of Medicine, Seoul, Korea; Wayne State University School of Medicine, UNITED STATES

## Abstract

Overexpression of p53 is the most frequent genetic alteration in breast cancer. Recently, many studies have shown that the expression of mutant p53 differs for each subtype of breast cancer and is associated with different prognoses. In this study, we aimed to determine the suitable cut-off value to predict the clinical outcome of p53 overexpression and its usefulness as a prognostic factor in each subtype of breast cancer, especially in luminal A breast cancer. Approval was granted by the Institutional Review Board of Samsung Medical Center. We analyzed a total of 7,739 patients who were surgically treated for invasive breast cancer at Samsung Medical Center between Dec 1995 and Apr 2013. Luminal A subtype was defined as ER&PR + and HER2- and was further subclassified according to Ki-67 and p53 expression as follows: luminal A (Ki-67-,p53-), luminal A (Ki-67+, p53-), luminal A (Ki-67 -, p53+) and luminal A (Ki-67+, p53+). Low-risk luminal A subtype was defined as negative for both Ki-67 and p53 (luminal A [ki-67-, p53-]), and others subtypes were considered to be high-risk luminal A breast cancer. A cut-off value of 10% for p53 was a good predictor of clinical outcome in all patients and luminal A breast cancer patients. The prognostic role of p53 overexpression for OS and DFS was only significant in luminal A subtype. The combination of p53 and Ki-67 has been shown to have the best predictive power as calculated by the area under curve (AUC), especially for long-term overall survival. In this study, we have shown that overexpression of p53 and Ki-67 could be used to discriminate low-risk luminal A subtype in breast cancer. Therefore, using the combination of p53 and Ki-67 expression in discriminating low-risk luminal A breast cancer may improve the prognostic power and provide the greatest clinical utility.

## Introduction

Hormone receptor (HR)-positive and HER2-negative breast cancer which is classified as luminal A breast cancer, generally shows favorable prognosis. However, some patients suffer from late recurrence. Given the clinical and molecular heterogeneity of luminal A breast cancer, there is a limited understanding of the mechanisms underlying treatment resistance and late relapse [1]. To identify the low-risk luminal A subtype, St. Gallen suggested the reclassification of luminal A breast cancer based on Ki-67 expression [2]. Recently, PR expression has been considered as another criteria for distinguishing the luminal A subtype [3].

p53 is the main regulator of genomic stability through regulation of the cell cycle. Overexpression of p53, which is caused by TP 53 mutation, is the most frequent genetic alteration in breast cancer [4, 5]. Recently, many studies have shown that the expression of p53 mutations differs for each subtype [6] and is related to treatment resistance [7–9]. However, despite the high incidence of genetic alterations in breast cancer, there is no consensus concerning the clinical role of p53 overexpression or even potential clinical applications.

Therefore, in this study, we aimed to determine a suitable cut-off value to discriminate the clinical meaning of p53 overexpression and its usefulness as a prognostic factor in each subtype of breast cancer, especially in luminal A breast cancer.

## Patients and Methods

### Clinicopathologic characteristics

Approval was granted by the Institutional Review Board of Samsung Medical Center (IRB file No. 2014-09-047). To protect the personal information, patient records/information was anonymized and de-identified prior to analysis. We retrospectively reviewed the clinicopathologic records of patients diagnosed with surgically treated invasive breast cancer at Samsung Medical Center between Dec 1995 and Apr. 2013. During these periods, 7739 patients with complete pathologic data, including tumor size, nuclear grade, multiple tumors, the presence of lymphovascular invasion (LVI), TNM stage, and the expression of estrogen receptor (ER), progesterone receptor (PR) and HER2, Ki-67 and p53 were included in the analysis. To assess the role of p53 and Ki-67 in luminal A, data for Ki-67 was used from Mar 2003.

### Immunohistochemsistry (IHC) staining

Immunohistochemical staining was performed for ER, PR, HER 2, Ki-67 and p53 after surgery for each patient. The Allred score was used to evaluate ER and PR status [10]. The proportion and intensity scores were summed, and tumor cells with a total score of 3–8 were considered ER and PR positive. Scores of 0 and 1 according to IHC were considered negative for HER2 expression and a score of 3 was noted as positive. In the cases of HER2 grade II, HER2 data from FISH assays were recorded. The hot spots of Ki-67 staining (DAKO, clone MIB-1, dilution 1:300) in the cancer cells were counted using a computerized image analysis system (I-SOLUTION DT, Vancouver, British Columbia, Canada). Fourteen percent or more was considered as positive as suggestion of St.Gallen [2]. The IHC for p53 was performed using a mouse monoclonal anti-human p53 (clone:BP53.12) antibody (Invitrogen/MD21704USA) at 1:4000 dilution and an the autoimmunostainer (Leica Bond Polymer Refine detection kit/Leica Bond-Max staining system). For assessment of the positivity of immunostaining for each section, only nuclear staining was regarded as positive. We counted tumor cells with clearly brown reaction products in nuclei by monitoring at least 1,000 tumor cells from more than five high power fields where positive cells were present at a relatively uniform density. Two observers evaluated staining results independently and differences in interpretation were resolved by consensus.

### Intrinsic subtype classification by IHC

Recently, several studies have reported that the absence of PR is associated with poor prognosis and proposed that PR-negative breast cancer be considered luminal B subtype [3, 11, 12]. Therefore, we classified the patients into four subtype: (luminal A (ER&PR+, HER2-), luminal B (ER+PR- or ER-PR+/HER2- or ER or PR +/HER2+), HER2 enriched (ER-/PR-/HER2+) and triple negative (ER-/PR-/HER2-). To determine the prognostic effect of Ki-67 and p53 expression in luminal A subtype, luminal A was then subclassified by Ki-67 and p53 expression: luminal A (Ki-67-,p53-), luminal A (Ki-67+, p53-), luminal A (Ki-67 -, p53+) and luminal A (Ki-67+, p53+). Low-risk luminal A subtype was defined as negative for both Ki-67 and p53 (luminal A (ki-67-, p53-) and high-risk luminal A subtype was positive for Ki-67 and/or p53 (luminal A (Ki-67+, p53-), luminal A (Ki-67 -, p53+) and luminal A (Ki-67+, p53+)).

### Statistical analysis

Comparisons of clinicopathologic characteristics between groups were performed using Chi-square and Fisher’s exact tests for categorical variables. Cox’s proportional hazards model was used for univariable and multivariable analyses of prognostic values. To reduce the risk of multicollinearity, some of the closely correlated variables were excluded (TM vs. BCS and radiotherapy, stage and chemotherapy). The strength of the association between these factors were calculated with Cramer's V [13]. Estimation of overall survival (OS) and disease-free survival (DFS) was performed using the Kaplan–Meier method and differences between survival curves were assessed using the log-rank test. The time-dependent receiver operating characteristic (ROC) curve was estimated using the Kaplan-Meier method [14] and the area under the ROC curve (AUC) was calculated from the ROC curves. The optimal cut-off was chosen as the point with the most significant log-rank P-value for all possible cut-off points. To identify significant clinical factors with Cox’s proportional hazard model, we considered stepwise selection with Akaike’s Information Criterion (AIC). Statistical analysis was executed using SAS version 9.4 (SAS Institute, Cary, NC, USA) and R 3.0.2 (Vienna, Austria; http://www.R-project.org).

## Results

### Cut-off value of p53 overexpression to predict prognosis (Fig 1)

**Fig 1:**
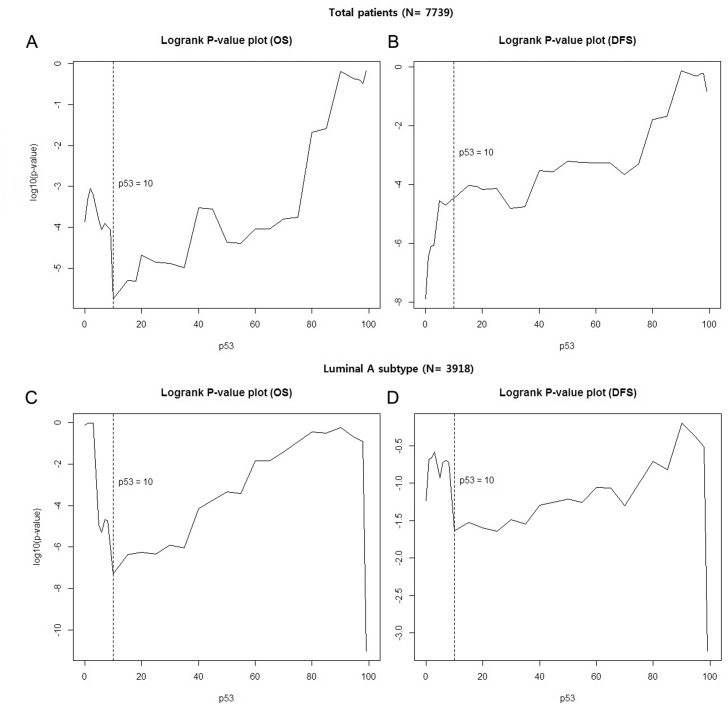
Cut-off value of p53 overexpression to predict the prognosis. (A and B) in all patients, and (C and D) in luminal A subtype.

To determine the prognostic role of p53, we first needed to identify the optimal cut-off level of p53 in breast cancer. We calculated the log-rank P-value to choose the optimal cut-off for overall survival (OS) and disease-free survival (DFS). The lowest P-value to predict OS was shown at 10%. For DFS, 35% was the most significant cut-off value; however, 10% of p53 overexpression was also significant (P = 0.000033). The cut-off value for the best predictor in luminal A subtype was also 10% for OS and DFS. Based on these results, we defined negative as absent or ≤10% nuclear staining and positive as > 10% nuclear staining.

### The association between overexpression of p53 and clinicopathologic characteristics

The median patient age was 48 years (range 21–90). The median level of Ki-67 was 20.38% (range 0.27–99.86). p53 overexpression was statistically associated with aggressive clinicopathologic features, including higher nuclear grade, advanced pathologic stage, ER and PR negativity and positive HER2 expression. p53 overexpression was most common in triple negative breast cancer (TNBC) subtype (56.3% (741/1315)) (Table 1).

**Table 1:** Clinicopathologic characteristics of all patients according to expression of p53 (N = 7739).

		Total (N = 7739)	p53 expression		P-value
			Negative (N = 5512)	Positive (N = 2227)	
Breast surgery					0.221
	TM	2715 (35.1)	1957 (35.5)	758 (34.0)	
	BCS	5024 (64.9)	3555 (64.5)	1469 (66.0)	
Presence of LVI					0.018
	No	5575 (72.0)	4013 (72.8)	1562 (70.1)	
	Yes	2164 (28.0)	1499 (27.2)	665 (29.9)	
RM					0.874
	Negative	7576 (97.9)	5395 (97.9)	2181 (97.9)	
	Positive	163 (2.1)	117 (2.1)	46 (2.1)	
NG					<0.0001
	1 & 2	4669 (60.3)	3946 (71.6)	723 (32.5)	
	3	3070 (39.7)	1566 (28.4)	1504 (67.5)	
AJCC Stage					<0.0001
	1	3285 (42.4)	2457 (44.6)	828 (37.2)	
	2	3398 (43.9)	2321 (42.1)	1077 (48.4)	
	3	1056 (13.6)	734 (13.3)	322 (14.5)	
ER					<0.0001
	Negative	2433 (31.4)	1100 (20.0)	1333 (59.9)	
	Positive	5306 (68.6)	4412 (80.0)	894 (40.1)	
PR					<0.0001
	Negative	2894 (37.4)	1449 (26.3)	1445 (64.9)	
	Positive	4845 (62.6)	4063 (73.7)	782 (64.9)	
HER2					<0.0001
	Negative	5542 (71.6)	4257 (77.2)	1285 (57.7)	
	Positive	2197 (28.4)	1255 (22.8)	942 (42.3)	
Molecular subtype					<0.0001
	Luminal A	3918 (50.6)	3468 (62.9)	450 (20.2)	
	Luminal B	1492 (19.3)	995 (18.1)	497 (22.3)	
	HER2-enriched	1014 (13.1)	475 (8.6)	539 (24.2)	
	TNBC	1315 (17.0)	574 (10.4)	741 (33.3)	

BCS, breast-conserving surgery; ER, estrogen receptor; IDC, invasive ductal carcinoma; ILC, invasive lobular carcinoma; LVI, lymphovascular invasion; NG, nuclear grade; PR, progesterone receptor; RM, resection margin; TM, total mastectomy; TNBC, triple negative breast cancer.

**Fig 2:**
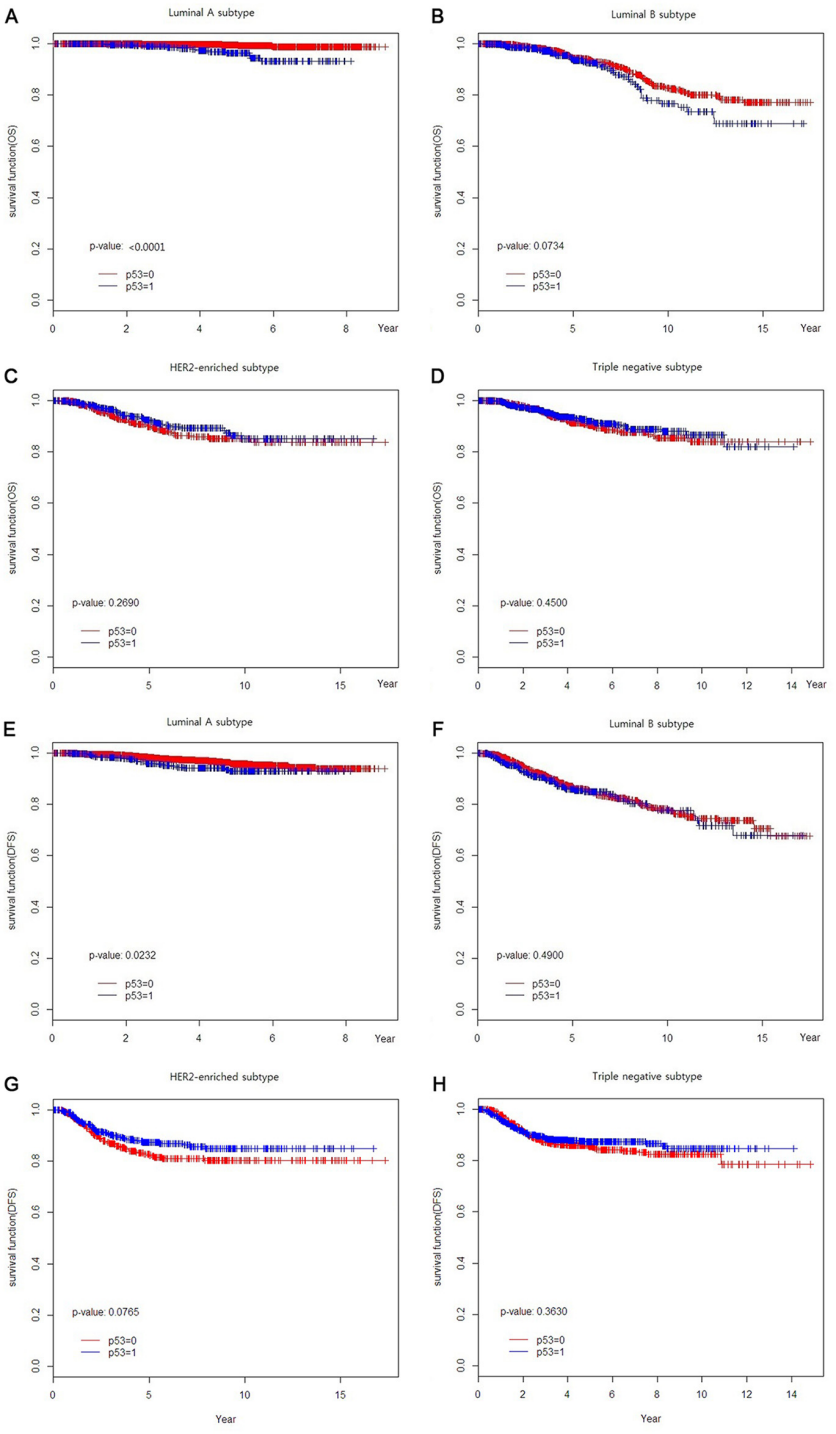
Clinical outcomes of each subtype according to p53 expression (A-D) Overall survival, (E-H) Disease-free survival.

From a total of 7739 patients, 3918 (50.6%), 1492 (19.3%), 1014 (13.1%) and 1315 (17.0%) were classified as luminal A, luminal B, HER2-enriched and TNBC subtypes, respectively. We stratified each subtype by p53 expression and analyzed survival according to p53 expression. Among the four subtypes, the luminal A subtype was only affected by p53 overexpression in OS and DFS (P < 0.0001 and P = 0.0232, respectively) (Fig 2).

**Fig 3:**
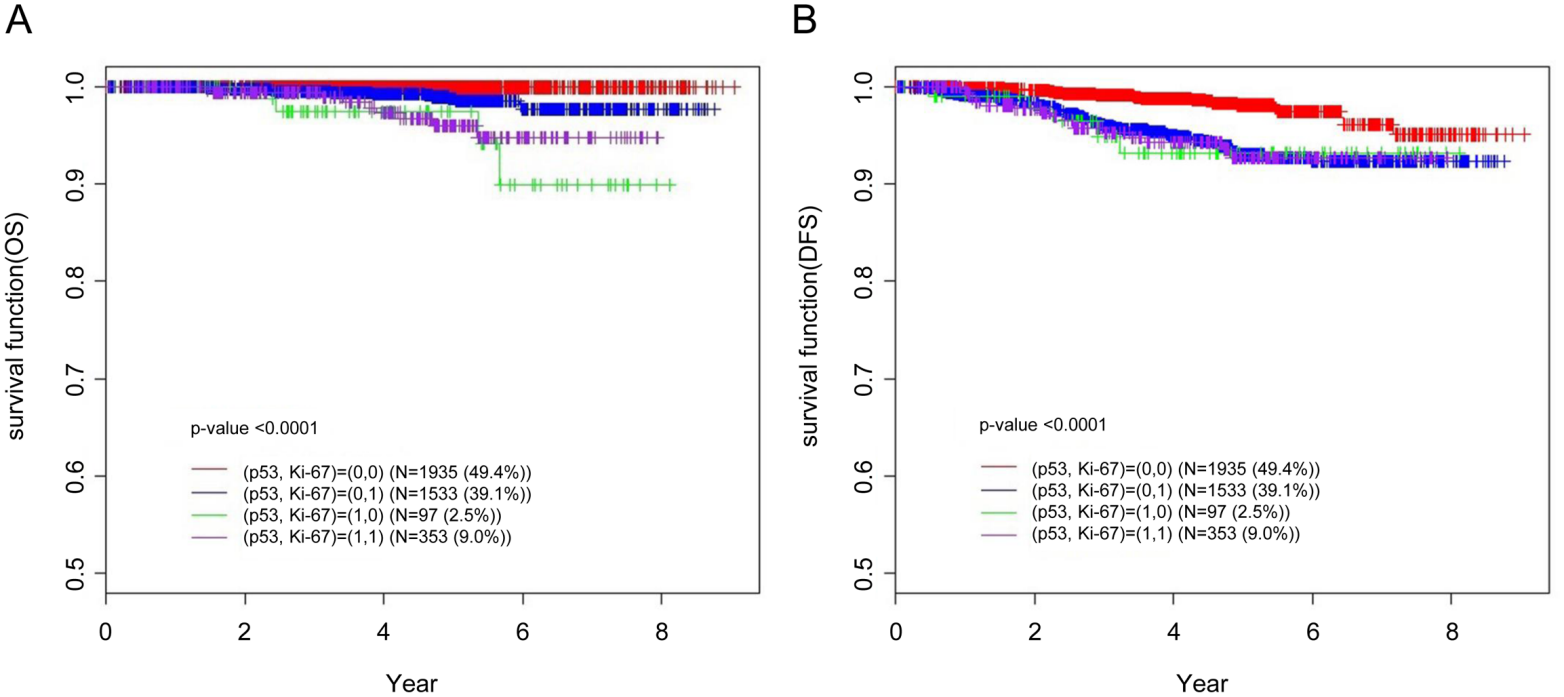
Clinical outcomes of luminal A breast cancer stratified according to p53 and Ki-67 expression (A) overall survival, (B) disease-free survival.

In this study, the prognostic role of p53 overexpression was only significant in luminal A subtype. As suggested by St.Gallen [2], we re-classified luminal A subtype according to Ki-67 expression. In addition to Ki-67 expression, we evaluated the prognostic role of 53 overexpression in luminal A subtype. Luminal A was subclassified into luminal A (Ki-67-,p53-), luminal A (Ki-67-, p53+), luminal A (Ki-67 +, p53-) and luminal A (Ki-67+, p53+). With the addition of one more criterion (p53 overexpression), we determined the low-risk luminal A subtype that showed the best clinical outcome to be that not expressing either Ki-67 or p53 (luminal A (Ki-67-, p53-) (Fig 3).

### Prognostic role of Ki-67 and p53 overexpression with regard to disease progression in luminal A breast cancer

**Fig 4:**
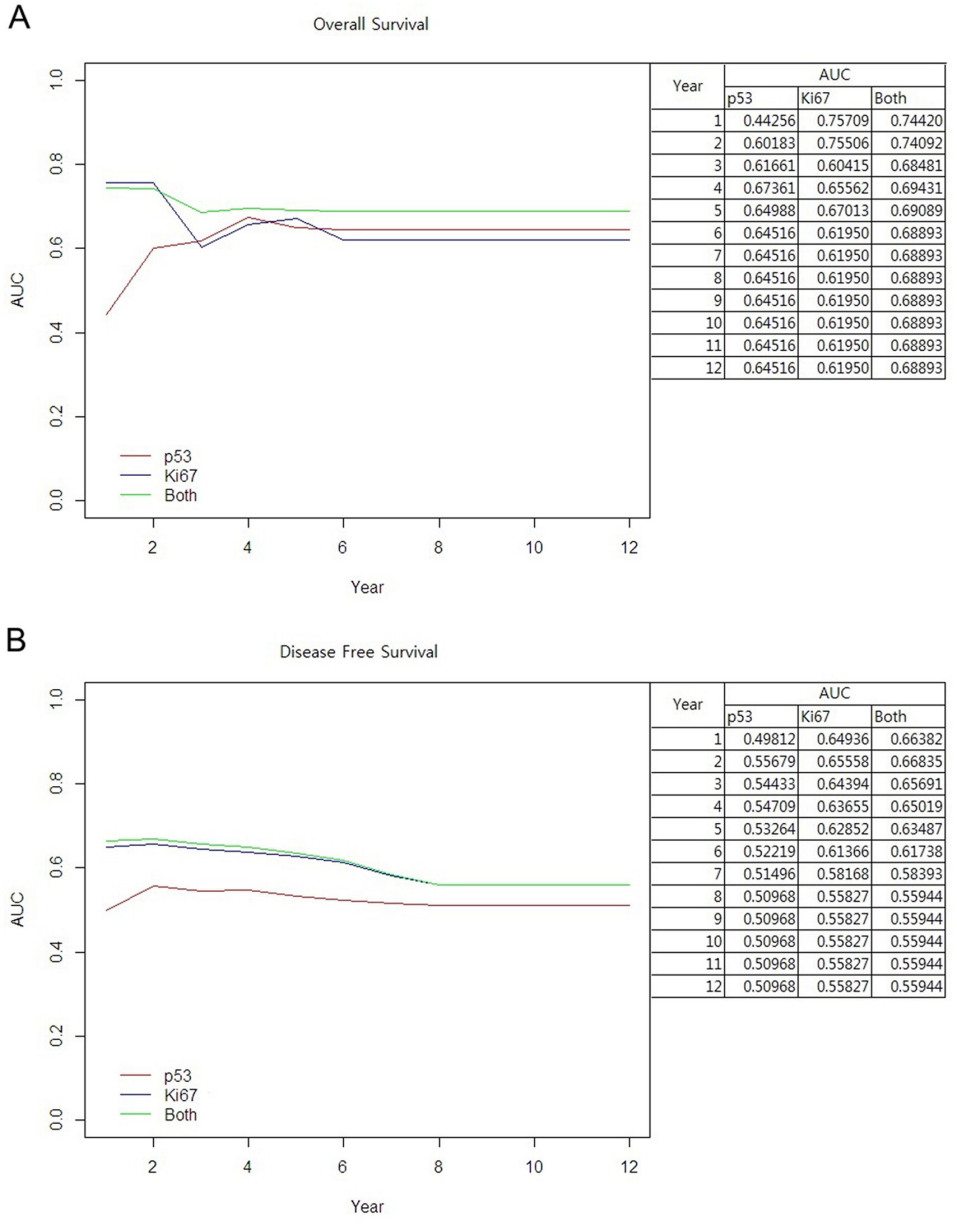
AUC curve over time. (A) overall survival, (B) disease-free survival.

We calculated the predictive power of Ki-67, p53 and the combination (Ki-67 and p53) with the area under curve (AUC) with regard to disease progression in luminal A breast cancer. In the prediction of OS, the combination of Ki-67 and p53 showed the best performance, especially for long-term survival. The predictive power of p53 alone or in combination with Ki-67 was superior to that of Ki-67 alone for long-term overall survival (Fig 4A). In the prediction of DFS, the combination of Ki-67 and p53 was superior to that of either p53 or Ki-67 alone; however, the difference in AUC between Ki-67 alone or in combination with p53 was very small. This indicates that Ki-67 alone is a good predictor of DFS (Fig 4B).

To know the clinicopathologic factors which predict the prognosis in luminal A subgroup, we performed univariable analysis with Cox-regression analysis. Performing total mastectomy, presence of LVI, higher nuclear grade, high pathologic stage, expression of Ki-67 and p53 were associated with poor outcomes (OS and DFS) (Tables 2 and 3) in univariable analysis. Performing chemotherapy and radiotherapy were not associated with OS. However, performing chemotherapy raised the recurrence ironically in univariable analysis (Table 3). It was probably because of high incidence of chemotherapy in high stage disease. Because endocrine therapy is standard treatment in the luminal A subgroup, there were only 17 patients who did not receive endocrine therapy. Cox-regression analysis of performing endocrine therapy in OS was not possible because there were no events in the group of no endocrine therapy. Endocrine therapy did not affect recurrence (Table 3).

**Table 2:** Factors associated with overall survival in luminal A subtype breast cancer patients (N = 3918).

			Univariable			Multivariable*	
Factors		HR	P-value	95% CI for HR	HR	P-value	95% CI for HR
Breast surgery							
	TM	- (ref.)	- (ref.)	- (ref.)			
	BCS	0.443	**0.0318**	0.211–0.932			
Presence of LVI							
	No	- (ref.)	- (ref.)	- (ref.)			
	Yes	4.783	**<0.0001**	2.163–10.578			
RM							
	Positive	2.726	0.3269	0.367–20.244			
	Negative	- (ref.)	- (ref.)	- (ref.)			
NG							
	1&2	- (ref.)	- (ref.)	- (ref.)			
	3	3.793	**0.0004**	1.806–7.969			
AJCC Stage							
	1	- (ref.)	- (ref.)	- (ref.)	- (ref.)	- (ref.)	- (ref.)
	2	2.283	0.1697	0.703–7.416	1.784	0.3380	0.546–5.833
	3	12.967	**<0.0001**	4.303–39.083	9.912	**<0.0001**	3.265–30.096
Ki-67	Positive	4.294	**0.0032**	1.631–11.303	2.587	0.0603	0.960–6.971
	Negative	- (ref.)	- (ref.)	- (ref.)	- (ref.)	- (ref.)	- (ref.)
p53	Positive	6.107	**<0.0001**	2.905–12.837	4.494	**<0.0001**	2.105–9.594
	Negative	- (ref.)	- (ref.)	- (ref.)	- (ref.)	- (ref.)	- (ref.)
**Chemotherapy** **	Yes	1.186	0.6989	0.50–2.815			
	No	- (ref.)	- (ref.)	- (ref.)			
**Radiotherapy** **	Yes	0.715	0.455	0.296–1.725			
	No	- (ref.)	- (ref.)	- (ref.)			
Anti-hormonal therapy** ^‡^							

BCS, breast-conserving surgery; LVI, lymphovascular invasion; NG, nuclear grade; RM, resection margin; TM, total mastectomy.

* Cox-proportional hazard regression model.

** A total of 3863 patients were included in this analysis.

^‡^ There was no event in the group of no anti-hormonal therapy. Therefore, Cox regression analysis was not performed.

**Table 3:** Factors associated with disease-free survival in luminal A subtype breast cancer patients (N = 3918).

			Univariable			Multivariable*	
Factors		HR	P-value	95% CI for HR	HR	P-value	95% CI for HR
Breast surgery							
	TM	- (ref.)	- (ref.)	- (ref.)	- (ref.)	- (ref.)	- (ref.)
	BCS	0.321	**<0.001**	0.26–0.538	0.567	**0.012**	0.364–0.882
Presence of LVI							
	No	- (ref.)	- (ref.)	- (ref.)	- (ref.)	- (ref.)	- (ref.)
	Yes	5.73	**<0.001**	2.95–6.307	2.195	**0.003**	1.312–3.671
RM							
	Positive	2.146	0.195	0.766–5.654			
	Negative	- (ref.)	- (ref.)	- (ref.)			
NG							
	1&2	- (ref.)	- (ref.)	- (ref.)	- (ref.)	- (ref.)	- (ref.)
	3	3.342	**<0.001**	1.997–4.177	1.401	0.149	0.886–2.215
AJCC Stage							
	1	- (ref.)	- (ref.)	- (ref.)	- (ref.)	- (ref.)	- (ref.)
	2	5.777	**<0.0001**	2.111–6.428	3.394	**0.004**	1.475–7.809
	3	22.428	**<0.0001**	6.115–19.079	8.535	**<0.0001**	3.549–20.522
Ki-67	Positive	3.798	**<0.0001**	1.909–4.381	2.466	**0.001**	1.443–4.213
	Negative	- (ref.)	- (ref.)	- (ref.)	- (ref.)	- (ref.)	- (ref.)
p53	Positive	1.777	**0.0335**	1.069–2.704	1.131	0.664	0.65–1.968
	Negative	- (ref.)	- (ref.)	- (ref.)	- (ref.)	- (ref.)	- (ref.)
**Chemotherapy** **	Yes	2.354	**0.0007**	1.438–3.854			
	No	- (ref.)	- (ref.)	- (ref.)			
**Radiotherapy** **	Yes	0.510	**0.001**	0.346–0.753			
	No	- (ref.)	- (ref.)	- (ref.)			
**Anti-hormonal therapy** **	Yes	0.087	0.055	0.020–1.041			
	No	- (ref.)	- (ref.)	- (ref.)			

BCS, breast-conserving surgery; LVI, lymphovascular invasion; NG, nuclear grade; RM, resection margin; TM, total mastectomy.

* Cox-proportional hazard regression model.

** A total of 3863 patients were included in this analysis.

**Fig 5:**
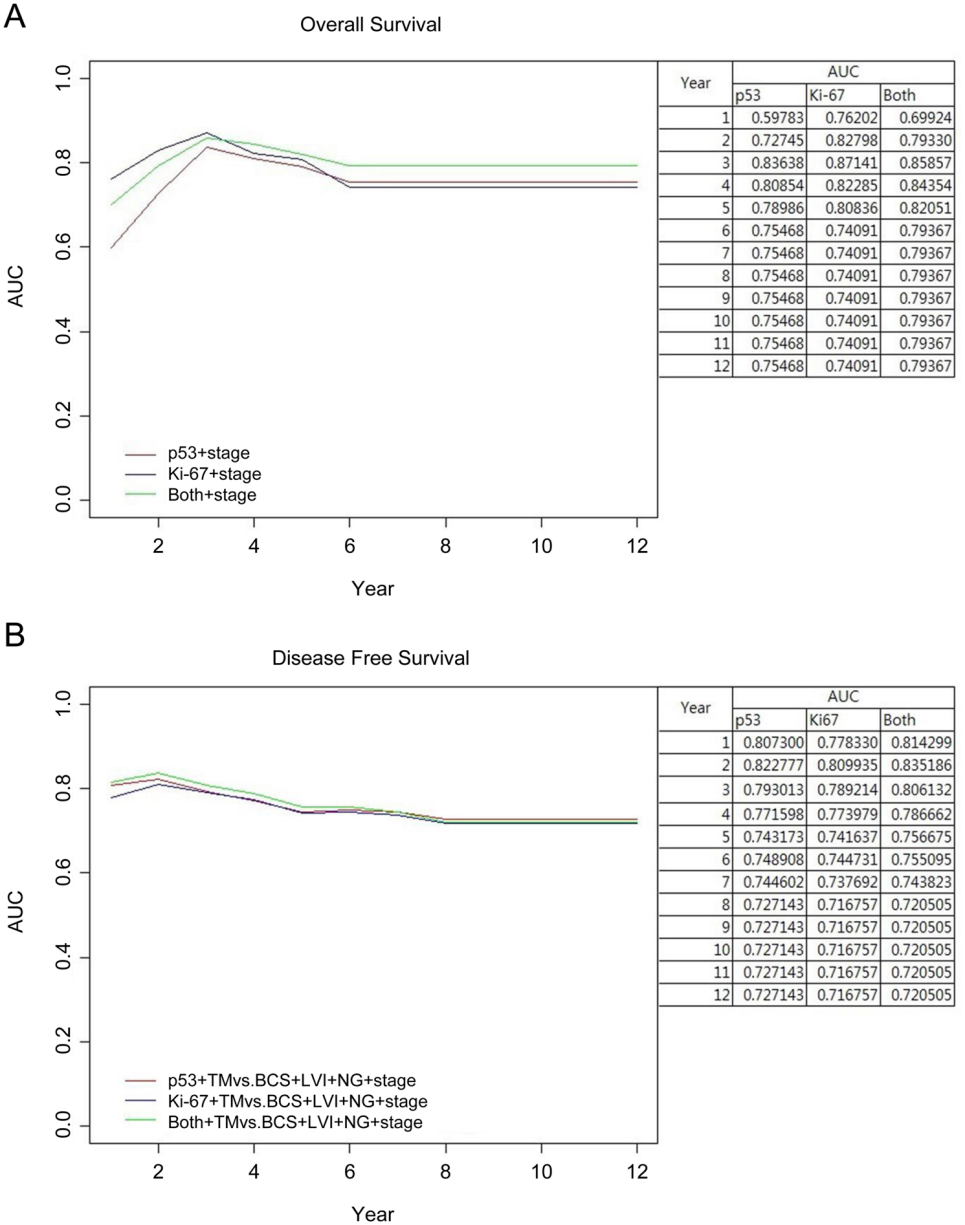
Adjusted AUC curve over time. (A) overall survival, (B) disease-free survival.

We performed multivariable Cox analysis with stepwise selection to test the usefulness of p53 and Ki-67 and found pathologic factors to be another predictor of OS and DFS. To reduce the risk of multicollinearity, some of the closely correlated variables were excluded for multivariable analysis. The strength of the association between these factors were calculated with Cramer's V [13]. The score of Cremer’s V were 0.7670 (operation methods and radiotherapy) and 0.6081 (stage and chemotherapy). These values imply strong associations between two factors [13]. The final model for OS included pathologic stage, Ki-67 and p53 overexpression. The other factors (NG, LVI and total mastectomy) that were significant in univariable analysis were statistically correlated with each other but were not significant in multivariable Cox analysis (Table 2). The values of AUC over time improved after accounting for disease stage (Fig 5A). After four years of follow-up, the predictive power of the combined measure was better than that of Ki-67 alone (Fig 5A). For DFS, Ki-67, p53, type of operation (TM vs. BCS), presence of LVI, NG and pathologic stage were included in multivariable Cox analysis (Table 3). Overexpression of p53 was not superior to Ki-67 expression in the prediction of DFS (Fig 5B).

## Discussion

In the present study, we identified a prognostic role of p53 overexpression in luminal A (ER+/PR+/HER2-) subtype. With Ki-67 and p53 overexpression, we discriminated the lower risk group of luminal A subtype, which showed the best outcomes. In addition to Ki-67, including p53 overexpression in luminal A breast cancer analysis was more predictive of overall survival, especially long-term survival.

Based on our data, we considered a positive result to be a greater than 10% nuclear staining with p53. With this cut-off value, 28.8% (2227/7739) of total patients and 11.5% (450/2918) of luminal A subtype patients showed p53 overexpression. There have been several previous studies reporting various definitions and rates of overexpression of p53. Some reports have used a scoring system [15–17], positive staining of mutant p53 in a single cancer cell [18] or various cut-off points based on the percent of immunohistochemical staining [8, 12, 19–22]. The reported p53 overexpression rate ranged widely from 14% to 44% [8, 12, 20, 21]. Many studies have used a p53 cut-off value of 10% [8, 12, 19, 20, 22], but few studies provide a reason for the chosen cut-off point. Therefore, in this study, we determined the proper cut-off value to predict the clinical outcomes in 7739 breast cancer patients. Concordant with results from other studies, we observed that the best clinical prediction value was at a p53 overexpression rate of 10% (lowest P-value for OS and significant DFS) (Fig 1). We also determined the best cut-off value in luminal A breast cancer. Kikuchi et al. reported a 50% cut-off value of p53 overexpression in luminal A subtype [21]. In our study, 10% was a cut-off value for best predictive value in luminal A subtype breast cancer.

Overexpression of p53 has been associated with aggressive features of tumor-like presence such as LVI, high nuclear grade, advanced stage, negative HR status and positive HER2 expression (Table 1). Many studies have shown similar results [8, 11, 12, 15, 18, 21–24].

The prognostic significance of p53 overexpression in breast cancer has been reported in several studies [12, 15–17, 22]. In the present study, p53 overexpression was related to poor prognosis in luminal A subtype but not in other subtypes. Many studies have reported that p53-overexpressing luminal subtype breast cancer showed significantly poor prognosis [8, 9, 11, 12, 17, 19, 21, 23]. The prognostic role of p53 overexpression has been shown in diverse clinical settings; pre- [12, 24] and post-menopausal [23] and node-negative [11] and node-positive [20] patients, neoadjuvant setting [22] and metastatic breast cancer [17]. The role of p53 overexpression as a predictive factor in breast cancer has also been suggested. Several studies have suggested that there is a relationship between p53 overexpression and endocrine therapy resistance and/or higher chemotherapy sensitivity [8, 9, 12, 17, 19, 22]. However, Lara et al. [20] showed that p53 overexpression was not a useful predictor of benefit from doxorubicin dose escalation or the addition of paclitaxel in node-positive patients. The poor outcomes of p53-expressing luminal A breast cancer in our study also supports a role of endocrine therapy resistance due to p53 overexpression.

Approximately 60–70% of human breast cancers express hormone receptors (HRs). Although HR-positive breast cancers classified as luminal subtype generally show favorable prognosis, this subtype also often shows late recurrence [25]. Luminal A breast cancer is a molecularly and clinically heterogeneous disease; nonetheless, there is a limited understanding of the mechanisms underlying treatment resistance and late relapse [1]. Recently, there have been several efforts to identify low- and high-risk HR-positive breast cancer. Gene tests like MammaPrint, Oncotype DX and PAM50 offer further assessment of risk of recurrence and help in determining the best treatment plan in breast cancer. However, molecular classification for clinical decision-making is limited by high cost. Many researchers have shown a close correlation between the genetic test and the established prognostic marker. Higher Oncotype Dx scores have been shown to be related to high-grade tumor, high Ki-67, negative PR and positive HER2 expression [26–29]. Cuzick et al. also suggested a new score system (IHC4 score) with an immunohistochemical panel of ER, PR, HER2 and Ki67 in conjunction with standard clinicopathological parameters. This score system provides similar prognostic information to the recurrence score in HR+ breast cancer treated with endocrine therapy [30]. Therefore, in practice, instead of expensive genetic testing, immunohistochemical panels could be used as surrogates to identify intrinsic breast cancer subtype. Traditionally, breast cancers are classified by ER, PR and HER2 expression into four subtypes; luminal A, luminal B, HER2-enriched and TNBC. Among the subtypes, luminal A subtype was re-classified with Ki-67 expression as suggested by St. Gallen [2]. Recently, PR expression was considered to be another criterion for distinguishing the luminal A subtype [3]. In the present study, we defined the luminal subtype based on the expression of both ER and PR. In addition to the prognostic role of Ki-67 and PR, we observed an additional role of p53 overexpression in distinguishing low-risk luminal A subtype.

In the present study, we showed that the combined expression of p53 and Ki-67 was better at discriminating the low-risk luminal A subtype than was expression of Ki-67 or p53 alone (Figs 4 and 5). Several studies have also shown that using overexpression of Ki67 in conjunction with that of p53 is prognostically informative in luminal A subtype breast cancer [11, 17, 19]. We investigated the role of p53 overexpression in luminal A subtype according to disease progression. We found that Ki-67 expression predicted overall survival throughout the course of disease, especially during early periods. The value of AUC increased further when Ki-67 was used in conjunction with p53 (Fig 4). As shown in Fig 4, the predictive power of the combined parameters was still superior to each individually after three years. With TNM staging, which is the traditional predictor, the AUC level increased to 0.8 (Fig 5). This is clinically important for luminal A subtype because of the unique characteristics of this subtype. Unlike other breast cancer subtypes that recur early, usually within five years, the recurrence in luminal breast cancers occurs until late periods [25, 31]. Despite the high incidence of genetic alterations in breast cancer, there is no consensus concerning the clinical role of p53 overexpression. We focused our attention on the association between p53 overexpression and prognosis in luminal A subtype group. Distinguishing the low-risk luminal A group is very important. It would be clinically significant if we could discriminate the patients who might gain an advantage from the anti-hormonal therapy than the usage of chemotherapy with the conventional IHC methods, even in node-positive luminal A patients. Conversely, if we know the high risk luminal A subgroup, we could consider more intensive or longer adjuvant therapy. Several studies also suggested the relationship between p53 overexpression and endocrine therapy resistance and/or higher chemotherapy sensitivity which is in accordance with our results [8, 9, 12, 17, 19, 22]. We think that IHC of p53 overexpression is relatively easy and useful method to help the discrimination of low risk luminal A subtype.

There are some limitations to this study. In our results, we could see the different results of p53 overexpression in DFS and OS. It is probably because of characteristics of breast cancer which survive long even after recurrence. As seen in our results of p53 which predict the long-term results, therefore, p53 did not show the significance in disease free survival. However, we could not explain the exact reason. Another major limitation of this study was the assessment of p53 mutation by IHC. IHC shows the resulting protein accumulation from p53 mutations. Relying only on IHC staining may result in lack of detection of important activation or inactivation within the p53 gene. However, checking of p53 overexpression with IHC is relatively cheap and the use of this easy indicator is also an advantage of this study. The strengths of this study include the relatively large sample size and data completeness. Ahn et al. reported on the prognostic role of p53 overexpression in a large series of 10073 patients who were registered with the Korean Breast Cancer Registry System (KBCRS) [8]. As indicated by Ahn et al., one pitfall of their data was incompleteness because of the inherent limitation of using data from multiple institutes. In contrast to previous studies that do not show the reason for the cut-off value of p53 overexpression, we demonstrated the proper cut-off value and supported it with data. By analyzing the effect of p53 overexpression over time, we were able to discriminate the low-risk luminal A subtype that is predicted to have good long-term survival. To our knowledge, the present study is the first report suggesting a long-term prognostic effect of combined p53 overexpression and Ki-67 expression in luminal A subtype.

## Conclusion

In this study, we showed that the overexpression of p53 with Ki-67 could discriminate the low-risk luminal A subtype in breast cancer. Therefore, the combination of p53 and Ki-67 in discriminating low-risk luminal A breast cancer may improve the prognostic power and provide the greatest clinical utility.

## References

[1] Ciriello G, Sinha R, Hoadley KA, Jacobsen AS, Reva B, Perou CM, et al The molecular diversity of Luminal A breast tumors. Breast Cancer Res Treat. 2013;141: 409–420. 10.1007/s10549-013-2699-3 24096568 PMC3824397

[2] Goldhirsch A, Wood WC, Coates AS, Gelber RD, Thurlimann B, Senn HJ. Strategies for subtypes—dealing with the diversity of breast cancer: highlights of the St. Gallen International Expert Consensus on the Primary Therapy of Early Breast Cancer 2011. Ann Oncol. 2011;22: 1736–1747. 10.1093/annonc/mdr304 21709140 PMC3144634

[3] Prat A, Cheang MC, Martin M, Parker JS, Carrasco E, Caballero R, et al Prognostic significance of progesterone receptor-positive tumor cells within immunohistochemically defined luminal A breast cancer. J Clin Oncol. 2013;31: 203–209. 10.1200/JCO.2012.43.4134 23233704 PMC3532392

[4] Petitjean A, Mathe E, Kato S, Ishioka C, Tavtigian SV, Hainaut P, et al Impact of mutant p53 functional properties on TP53 mutation patterns and tumor phenotype: lessons from recent developments in the IARC TP53 database. Hum Mutat. 2007;28: 622–629. 17311302 10.1002/humu.20495

[5] Desmedt C, Voet T, Sotiriou C, Campbell PJ. Next-generation sequencing in breast cancer: first take home messages. Curr Opin Oncol. 2012;24: 597–604. 10.1097/CCO.0b013e328359554e 23014189 PMC3713550

[6] Dumay A, Feugeas JP, Wittmer E, Lehmann-Che J, Bertheau P, Espie M, et al Distinct tumor protein p53 mutants in breast cancer subgroups. Int J Cancer. 2013;132: 1227–1231. 10.1002/ijc.27767 22886769

[7] Ellis MJ, Perou CM. The genomic landscape of breast cancer as a therapeutic roadmap. Cancer Discov. 2013;3: 27–34. 10.1158/2159-8290.CD-12-0462 23319768 PMC3553590

[8] Ahn SH, Kim HJ, Han W, Cho J, Gong G, Jung KH, et al Effect modification of hormonal therapy by p53 status in invasive breast cancer. J Breast Cancer. 2013;16: 386–394. 10.4048/jbc.2013.16.4.386 24454460 PMC3893340

[9] Coutant C, Rouzier R, Qi Y, Lehmann-Che J, Bianchini G, Iwamoto T, et al Distinct p53 gene signatures are needed to predict prognosis and response to chemotherapy in ER-positive and ER-negative breast cancers. Clin Cancer Res. 2011;17: 2591–2601. 10.1158/1078-0432.CCR-10-1045 21248301

[10] Allred DC, Harvey JM, Berardo M, Clark GM. Prognostic and predictive factors in breast cancer by immunohistochemical analysis. Mod Pathol. 1998;11: 155–168. 9504686

[11] Feeley LP, Mulligan AM, Pinnaduwage D, Bull SB, Andrulis IL. Distinguishing luminal breast cancer subtypes by Ki67, progesterone receptor or TP53 status provides prognostic information. Mod Pathol. 2014;27: 554–561. 10.1038/modpathol.2013.153 24051696

[12] Kim HS, Yom CK, Kim HJ, Lee JW, Sohn JH, Kim JH, et al Overexpression of p53 is correlated with poor outcome in premenopausal women with breast cancer treated with tamoxifen after chemotherapy. Breast Cancer Res Treat. 2010;121: 777–788. 10.1007/s10549-009-0560-5 19806450

[13] Agresti A. Categorical data analysis. 2nd ed New York: Wiley-Interscience; 2002.

[14] Heagerty PJ, Lumley T, Pepe MS. Time-dependent ROC curves for censored survival data and a diagnostic marker. Biometrics. 2000;56: 337–344. 10877287 10.1111/j.0006-341x.2000.00337.x

[15] Friedrichs K, Gluba S, Eidtmann H, Jonat W. Overexpression of p53 and prognosis in breast cancer. Cancer. 1993;72: 3641–3647. 8252480 10.1002/1097-0142(19931215)72:12<3641::aid-cncr2820721215>3.0.co;2-8

[16] Hasebe T, Iwasaki M, Akashi-Tanaka S, Hojo T, Shibata T, Sasajima Y, et al p53 expression in tumor-stromal fibroblasts forming and not forming fibrotic foci in invasive ductal carcinoma of the breast. Mod Pathol. 2010;23: 662–672. 10.1038/modpathol.2010.47 20208478

[17] Yamashita H, Toyama T, Nishio M, Ando Y, Hamaguchi M, Zhang Z, et al p53 protein accumulation predicts resistance to endocrine therapy and decreased post-relapse survival in metastatic breast cancer. Breast Cancer Res. 2006;8: R48 16869955 10.1186/bcr1536 PMC1779473

[18] Marks JR, Humphrey PA, Wu K, Berry D, Bandarenko N, Kerns BJ, et al Overexpression of p53 and HER-2/neu proteins as prognostic markers in early stage breast cancer. Ann Surg. 1994;219: 332–341. 7909221 10.1097/00000658-199404000-00002 PMC1243148

[19] Millar EK, Graham PH, McNeil CM, Browne L, O'Toole SA, Boulghourjian A, et al Prediction of outcome of early ER+ breast cancer is improved using a biomarker panel, which includes Ki-67 and p53. Br J Cancer. 2011;105: 272–280. 10.1038/bjc.2011.228 21712826 PMC3142808

[20] Lara JF, Thor AD, Dressler LG, Broadwater G, Bleiweiss IJ, Edgerton S, et al p53 Expression in node-positive breast cancer patients: results from the Cancer and Leukemia Group B 9344 Trial (159905). Clin Cancer Res. 2011;17: 5170–5178. 10.1158/1078-0432.CCR-11-0484 21693655 PMC3149770

[21] Kikuchi S, Nishimura R, Osako T, Okumura Y, Nishiyama Y, Toyozumi Y, et al Definition of p53 overexpression and its association with the clinicopathological features in luminal/HER2-negative breast cancer. Anticancer Res. 2013;33: 3891–3897. 24023325

[22] Guarneri V, Barbieri E, Piacentini F, Giovannelli S, Ficarra G, Frassoldati A, et al Predictive and prognostic role of p53 according to tumor phenotype in breast cancer patients treated with preoperative chemotherapy: a single-institution analysis. Int J Biol Markers. 2010;25: 104–111. 20544688 10.1177/172460081002500208

[23] Yamamoto M, Hosoda M, Nakano K, Jia S, Hatanaka KC, Takakuwa E, et al p53 accumulation is a strong predictor of recurrence in estrogen receptor-positive breast cancer patients treated with aromatase inhibitors. Cancer Sci. 2014;105: 81–88. 10.1111/cas.12302 24118529 PMC4317887

[24] Talley L, Chhieng DC, Bell WC, Grizzle WE, Frost AR. Immunohistochemical detection of EGFR, p185(erbB-2), Bcl-2 and p53 in breast carcinomas in pre-menopausal and post-menopausal women. Biotech Histochem. 2008;83: 5–14. 10.1080/10520290701822436 18568671

[25] Early Breast Cancer Trialists' Collaborative Group. Effects of chemotherapy and hormonal therapy for early breast cancer on recurrence and 15-year survival: an overview of the randomised trials. Lancet. 2005;365: 1687–1717. 15894097 10.1016/S0140-6736(05)66544-0

[26] Asad J, Jacobson AF, Estabrook A, Smith SR, Boolbol SK, Feldman SM, et al Does oncotype DX recurrence score affect the management of patients with early-stage breast cancer? Am J Surg. 2008;196: 527–529. 10.1016/j.amjsurg.2008.06.021 18809056

[27] Flanagan MB, Dabbs DJ, Brufsky AM, Beriwal S, Bhargava R. Histopathologic variables predict Oncotype DX recurrence score. Mod Pathol. 2008;21: 1255–1261. 10.1038/modpathol.2008.54 18360352

[28] Mattes MD, Mann JM, Ashamalla H, Tejwani A. Routine histopathologic characteristics can predict oncotype DX(TM) recurrence score in subsets of breast cancer patients. Cancer Invest. 2013;31: 604–606. 10.3109/07357907.2013.849725 24164299

[29] Allison KH, Kandalaft PL, Sitlani CM, Dintzis SM, Gown AM. Routine pathologic parameters can predict Oncotype DX recurrence scores in subsets of ER positive patients: who does not always need testing? Breast Cancer Res Treat. 2012;131: 413–424. 10.1007/s10549-011-1416-3 21369717

[30] Cuzick J, Dowsett M, Pineda S, Wale C, Salter J, Quinn E, et al Prognostic value of a combined estrogen receptor, progesterone receptor, Ki-67, and human epidermal growth factor receptor 2 immunohistochemical score and comparison with the Genomic Health recurrence score in early breast cancer. J Clin Oncol. 2011;29: 4273–4278. 10.1200/JCO.2010.31.2835 21990413

[31] Kennecke H, Yerushalmi R, Woods R, Cheang MC, Voduc D, Speers CH, et al Metastatic behavior of breast cancer subtypes. J Clin Oncol. 2010;28: 3271–3277. 10.1200/JCO.2009.25.9820 20498394

